# Genome Sequencing Suggests Diverse Secondary Metabolism in Coral-Associated Aquimarina megaterium

**DOI:** 10.1128/mra.00620-22

**Published:** 2022-10-19

**Authors:** Joana Fernandes Couceiro, Tina Keller-Costa, Nikos C. Kyrpides, Tanja Woyke, William B. Whitman, Rodrigo Costa

**Affiliations:** a Institute for Bioengineering and Biosciences, Instituto Superior Técnico, Universidade de Lisboa, Lisbon, Portugal; b i4HB—Institute for Health and Bioeconomy, Instituto Superior Técnico, Universidade de Lisboa, Lisbon, Portugal; c Department of Bioengineering, Instituto Superior Técnico, Universidade de Lisboa, Lisbon, Portugal; d Department of Energy Joint Genome Institute, Lawrence Berkeley National Laboratory, Berkeley, California, USA; e Department of Microbiology, University of Georgia, Athens, Georgia, USA; f Centro de Ciências do Mar (CCMAR), Universidade do Algarve, Faro, Portugal; Montana State University

## Abstract

We report here the genome sequences of three Aquimarina megaterium strains isolated from the octocoral Eunicella labiata. We reveal a coding potential for versatile carbon metabolism and biosynthesis of natural products belonging to the polyketide, nonribosomal peptide, and terpene compound classes.

## ANNOUNCEMENT

Members of the genus *Aquimarina* (*Bacteroidetes, Flavobacteriaceae*) play important roles in carbon and nitrogen cycling in marine environments (1). They are also a rich reservoir of secondary metabolites with promising antimicrobial activities (1–3). Many *Aquimarina* species live in association with eukaryotic hosts, such as marine sponges (4), octocorals (5, 6), clams (7), or algae (8), although some strains are emerging pathogens, causing epizootic shell disease in crustaceans (9). Aquimarina megaterium is a strictly aerobic, nonflagellated bacterium that was first isolated from surface seawater in the South Pacific (10). Only one *A. megaterium* genome, that of planktonic type strain XH134, is currently available. Here, we report the genomes of three octocoral-associated *A. megaterium* strains isolated from Eunicella labiata in the northeast Atlantic.

The strains were isolated from one *E. labiata* specimen collected off the coast of Faro, Portugal (6). Host-derived microbial cell suspensions were plated on half-strength marine agar and incubated at 18°C for 1 week, and single colonies were streaked until purity on the same medium (6). *Aquimarina* isolates were identified by Sanger sequencing of 16S rRNA genes amplified from genomic DNA extracted from pure cultures freshly grown in marine broth, using the Wizard genomic DNA purification kit (Promega, USA) (6). The same DNA extracts were used for genome sequencing at the Joint Genome Institute (JGI) as part of the Genomic Encyclopedia of Type Strains Phase IV project. Default parameters were used for all software unless otherwise specified. Genome libraries (300 bp) were prepared with the KAPA HyperPrep kit (Kapa Biosystems) and sequenced using the Illumina NovaSeq 6000 platform (S4 flow cell). Raw reads were quality filtered per JGI standard operating practice (SOP) protocol 1061 using BBTools v.38.86 (http://bbtools.jgi.doe.gov). Filtered reads were assembled into contigs using SPAdes v.3.14.1 (11) with 25, 55, and 95 k-mers. Organism and project metadata were deposited in the Genomes OnLine database (12), and contigs were annotated using the NCBI Prokaryotic Genome Annotation Pipeline (PGAP v.6.2) (13) and DOE-JGI Microbial Genome Annotation Pipeline (MGAP v.4) (14). Results were submitted to the Integrated Microbial Genomes and Microbiomes system (IMG/M) for comparative analysis (15). Genome completeness and contamination were assessed with the Microbial Genomes Atlas (16). AntiSMASH v6.0 (17) was used to identify secondary metabolite biosynthetic gene clusters (SM-BGCs).

The general features of the genomes are provided in Table 1. Average nucleotide identities (ANIs) obtained on IMG/M (15) for octocoral-derived strains EL32, EL35, and EL43 and *A. megaterium* type strain XH134 were 99.06% or above, ascertaining their same-species status. Likewise, digital DNA-DNA hybridization (dDDH) probabilities (18) calculated for all strain combinations were above 97%.

**TABLE 1:** General features of the Aquimarina megaterium strain genomes reported in this study

Strain^*a*^	IMG ID^*b*^	Size (Mb)	GC (%)	Coverage	No. of contigs (*N*_50_)^*c*^	Read		Estimate (%) of:		No. of^*d*^:		Count by^*d*^:					Accession no.^*e*^
						Count	Length (bp)	Completeness	Contamination	Genes	CDSs	RNA	rRNA	tRNA	ncRNA	Pfam	
EL_32	2880550035	6.06	33.00	249.6×	44 (334,002)	22,409,030	151	97.2	0.9	5,331†|5,213*	5,266†|5,166*	46†|47*	3†|3*	40†|40*	3†|4*	3,842†	JADOUG000000000, GCF_015752005.1, SRR13202134
EL_35	2880555367	6.06	32.99	248.9×	46 (325,984)	27,572,010	151	97.2	0.9	5,330†|5,215*	5,265†|5,168*	46†|47*	3†|3*	40†|40*	3†|4*	3,843†	JADOUH000000000, GCF_015751855.1, SRR13202145
EL_43	2880762256	6.05	33.00	249.7×	48 (326,032)	28,518,382	151	97.2	0.9	5,321†|5,203*	5,256†|5,156*	46†|47*	3†|3*	40†|40*	3†|4*	3,839†	JADOUM000000000, GCF_015752065.1, SRR13202146

a All strains reported in this study have been isolated from the octocoral host Eunicella labiata.

b Unique genome identifier on the Integrated Microbiomes and Metagenomes (IMG/M) portal.

c Contig *N*_50_ metrics are provided in numbers of base pairs.

d Annotation was performed using the DOE-JGI Microbial Genome Annotation Pipeline (MGAP v.4) (†) and the NCBI Prokaryotic Genome Annotation Pipeline (Pgap v.6.2) (*).

e Accession numbers for GenBank, the assembly, and raw reads are listed in that order.

**FIG 1:**
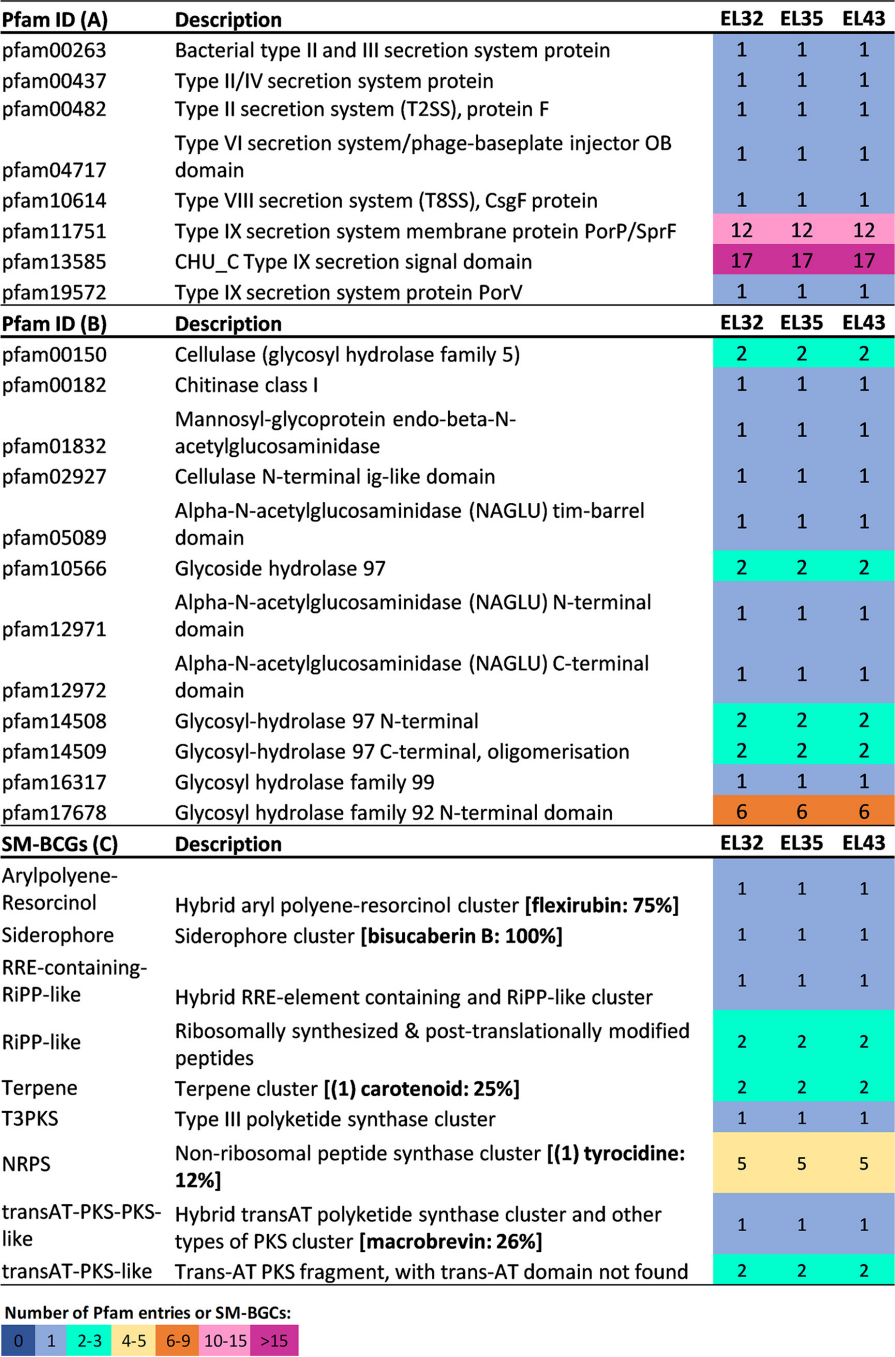
Presence and abundance of select functional features encoded by the Aquimarina megaterium genomes reported in this study. Pfam entries involved in protein secretion systems (A) and carbohydrate metabolism (B) are shown, along with SM-BGCs (C) identified with AntiSMASH v.6.0.

All three genomes contain genes encoding several glycoside hydrolases, feature cellulase-, chitinase-, and *N*-acetylglucosaminidase-encoding genes, and contain multiple domains related to the type IX secretion system involved in gliding motility and chitinase export (19) (Fig. 1), underpinning a versatile carbon metabolism (1). Moreover, all strains possess the potential to synthesize terpenes, polyketides, nonribosomal and ribosomal peptides, flexirubin-like pigments, and siderophores (Fig. 1), congruent with the notion of *Aquimarina* species as reservoirs of chemical diversity of biotechnological interest (1, 3).

### Data availability.

The genome sequences of the three Aquimarina megaterium strains have been deposited in GenBank/NCBI by the JGI. GenBank accession numbers are listed in Table 1.
